# An epithelial-to-mesenchymal transition-inducing potential of granulocyte macrophage colony-stimulating factor in colon cancer

**DOI:** 10.1038/s41598-017-08047-1

**Published:** 2017-08-15

**Authors:** Yaqiong Chen, Zhi Zhao, Yu Chen, Zhonglin Lv, Xin Ding, Renxi Wang, He Xiao, Chunmei Hou, Beifen Shen, Jiannan Feng, Renfeng Guo, Yan Li, Hui Peng, Gencheng Han, Guojiang Chen

**Affiliations:** 1 Department of Immunology, Institute of Basic Medical Sciences, Beijing, 100850 P.R. China; 2 College of Pharmacy, China Pharmaceutical University, Nanjing, 210009 P.R. China; 3 Department of Pathology, Yihe Hospital, Henan University, Zhengzhou, 450000 P.R. China; 4 Department of Experimental Animals, Zhejiang Academy of Traditional Chinese Medicine, Hangzhou, 310007 P.R. China; 5 Graduate School, Anhui Medical University, Hefei, 230032 P.R. China; 6 Department of Pathology, University of Michigan, Ann Arbor, MI 48109 USA; 7 Department of Environment and Pharmacy, Institute of Health and Environmental Medicine, Tianjin, 300050 P.R. China

## Abstract

Growing evidence shows that granulocyte macrophage colony-stimulating factor (GM-CSF) has progression-promoting potentials in certain solid tumors, which is largely attributed to the immunomodulatory function of this cytokine in tumor niches. However, little is known about the effect of GM-CSF on cancer cells. Herein, we show that chronic exposure of colon cancer cells to GM-CSF, which harbor its receptor, leads to occurrence of epithelial to mesenchymal transition (EMT), in time and dose-dependent manners. These GM-CSF-educated cancer cells exhibit enhanced ability of motility *in vitro* and *in vivo*. Furthermore, GM-CSF stimulation renders colon cancer cells more resistant to cytotoxic agents. Mechanistic investigation reveals that MAPK/ERK signaling and EMT-inducing transcription factor ZEB1 are critical to mediate these effects of GM-CSF. In specimen of CRC patients, high-level expression of GM-CSF positively correlates with local metastases in lymph nodes. Moreover, the co-expression of GM-CSF and its receptors as well as phosphorylated ERK1/2 are observed. Thus, our study for the first time identifies a progression-promoting function of GM-CSF in colon cancer by inducing EMT.

## Introduction

Granulocyte macrophage colony-stimulating factor (GM-CSF) is a hematopoietic growth factor produced by a variety of cell types, including macrophages, T lymphocytes, fibroblasts, endothelial cells, and keratinocytes following appropriate stimuli^1^. Recently, clinical data show that GM-CSF levels in the circulatory system were frequently elevated in patients suffering from colorectal cancer compared with healthy controls^2–6^. Furthermore, high GM-CSF expression in neoplastic lesions significantly correlated with local and distant metastasis, more advanced histological grade, and poor prognosis in patients with breast cancer^7^ and pancreatic ductal carcinoma^8, 9^. These disease-promoting effects of GM-CSF may be linked to modulation of immune reactions in tumor niche, involving macrophages^7, 10^, myeloid-derived suppressor cells^9, 11, 12^, and plasmacytoid predendritic cells^13^.

It is worthy to note that the receptors for GM-CSF are constitutively expressed on the surface of certain nonhematopoietic cancer cells^14, 15^, which suggests the possibility of responsiveness of cancer cells to GM-CSF challenge. Indeed, GM-CSF has been reported to support tumor growth and progression by autocrine stimulation of proliferation and migration in squamous cell carcinoma of skin or head and neck^16, 17^, gliomas^18^ and osteosarcoma^19^. Furthermore, it also enhances the invasive capacity of human cancer cells *in vitro* by increasing production and activation of several matrix metalloproteinases^20–22^. The mechanisms underlying the metastasis-promoting function of GM-CSF are still incompletely known and need to be further addressed.

Epithelial to mesenchymal transition (EMT) is a developmental process that seems to play an important role in tumor progression and metastasis in diverse solid tumors, including colorectal cancer^23^. The EMT phenotype is characterized by the loss of cell-to-cell adhesion with the disintegration of tight and gap junctions and a phenotypic change from an “epithelial” morphology to a motile, fibroblast-like morphology^24, 25^. The hallmark of EMT is the functional loss of E-cadherin, while additional cellular changes, such as reduced expression of epithelial markers cytokeratins and ZO-1, and the overexpression of mesenchymal markers N-cadherin, vimentin and fibronectin, are also often observed. Several transcription factors (TF) have been identified as master regulators of EMT, including the Snail, Zeb and Twist families, all of which interact with E-box elements located within the proximal region of the E-cadherin promoter^24^. Additionally, EMT-inducing TF expression is tightly regulated at different steps of transcription, translation and protein stability control by a variety of cell-intrinsic pathways as well as extracellular clues^26^.

In this study, we hypothesize that exogenous GM-CSF stimulation in colon cancer cells is competent to induce EMT and thereby enhance their motility *in vitro* and *in vivo*. Chronic treatment of GM-CSF or ectopic expression of GM-CSF led to the occurrence of EMT in receptor-expressing colon cancer cells, but not in receptor-negative counterparts. These GM-CSF-educated cancer cells displayed enhanced ability of migration and invasion *in vitro* and more metastatic nodules in a mouse model of colorectal liver metastasis. Mechanistically, MAPK/ERK signals and ZEB1 were required for GM-CSF-induced EMT phenotype. Furthermore, the exposure to GM-CSF rendered colon cancer cells more resistant to drug-mediated cell death.

## Results

### Chronic exposure to GM-CSF induces EMT in colon cancer cells

**Figure 1:**
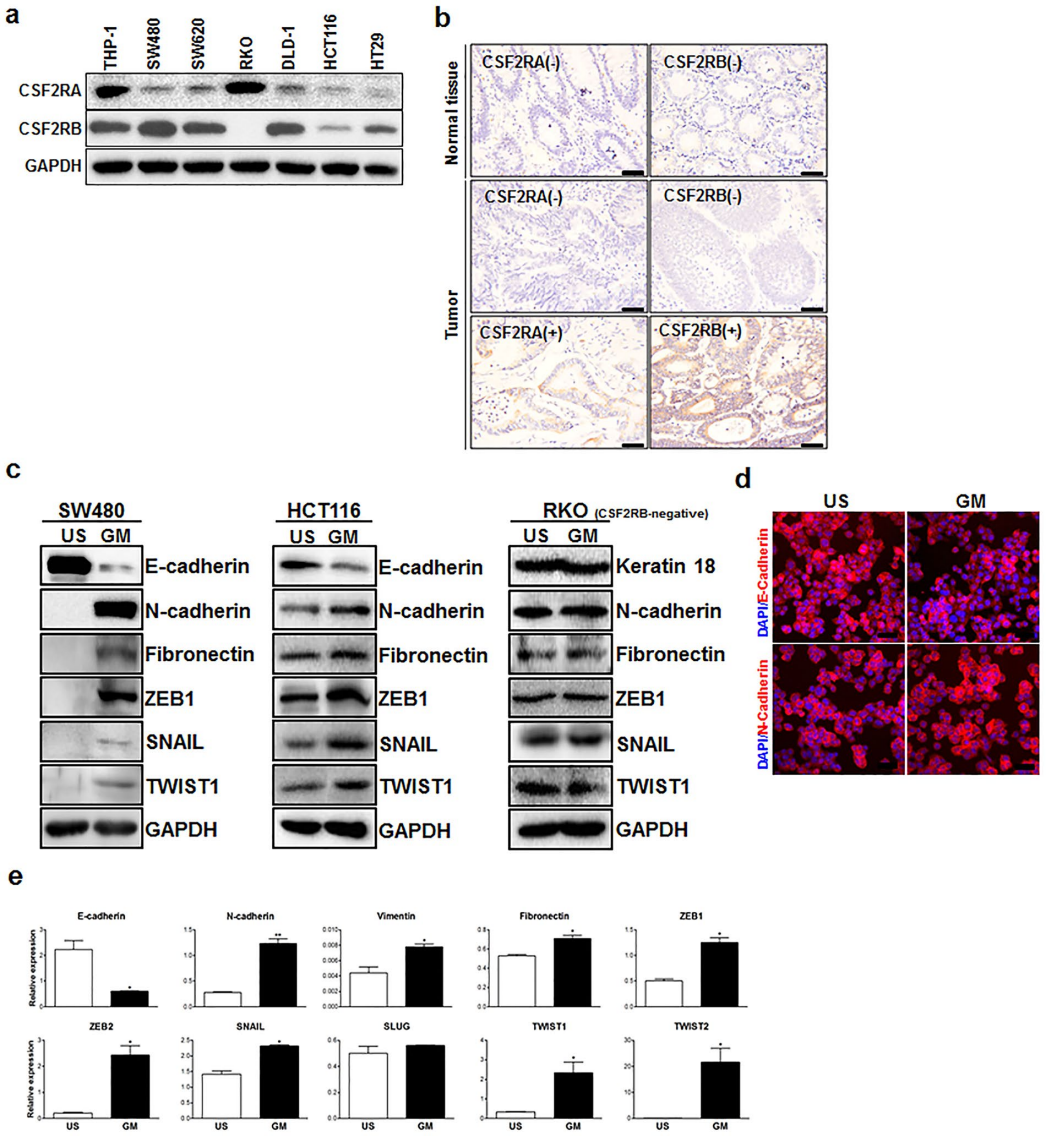
Chronic stimulation of colon cancer cells with GM-CSF leads to EMT. (**a**) The subunit α and β of GM-CSF receptor in six colon cancer cell lines were examined by Western blotting. A human monocytic cell line THP-1 was used as a positive control. (**b**) GM-CSF receptor was detected by IHC in specimen (tumor and adjacent normal colon tissues) of CRC patients. Representative data were shown. Scale bar: 50 μm. (**c**) Three colon cancer cell lines (SW480, HCT116 and RKO) were stimulated with GM-CSF (25 ng/ml) for three weeks. The expression of epithelial and mesenchymal markers as well as EMT-related transcriptional factors indicated was examined by immunoblotting. Representative data of cropped blots from five independent experiments were shown. (**d**) The expression of E-cadherin and N-cadherin in SW480 cell line treated with GM-CSF as described above was detected by immunofluorescence. Representative images from three independent experiments were shown. Scale bar: 100 μm. (**e**) SW480 cell line was treated with GM-CSF as described above. The expression of EMT-related markers and transcriptional factors was examined by quantitative RT-PCR. The data were pooled from three experiments. US: unstimulated; GM: GM-CSF. *P < 0.05; **P < 0.01 vs untreated controls.

Firstly, we detected the constitutive expression of the receptors for GM-CSF in colon cancer cells. Indeed, in agreement with our previous study^27^, five of six cell lines virtually harbored two GM-CSF receptor subunits A and B, albeit different abundance (Fig. 1a and S1a). Of note, RKO cell line was devoid of subunit B of GM-CSF receptor (Fig. 1a and S1a), which was pivotal for signaling transduction upon the engagement of GM-CSF and its receptor. This cell line, thus, was utilized as GM-CSF-nonresponsive counterparts. Furthermore, we also observed the expression of GM-CSF receptors in tumor cells of specimens from CRC patients (Fig. 1b), indicating a direct responsiveness of colon cancer cells to GM-CSF. Importantly, a three-week treatment of exogenous GM-CSF resulted in EMT program in three cell lines (SW480, HCT116 and HT29), which expressed two subunits of GM-CSF receptors as described above. Specifically, chronic exposure to GM-CSF remarkably repressed E-cadherin expression and simultaneously up-regulated the expression of mesenchymal markers N-cadherin and fibronectin (Fig. 1c–e and S1b). In accord with this, the levels of EMT-regulating master regulators including SNAIL and TWIST1 were elevated dramatically following GM-CSF stimulation (Fig. 1c and e and S1b). Notably, the up-regulation of ZEB1 levels was most significant (Fig. 1c and S1b). Furthermore, the EMT-inducing effects of GM-CSF were time and dose-dependent (Fig. S1c). Intriguingly, GM-CSF stimulation did not affect the expression of epithelial and mesenchymal markers in GM-CSF-nonresponsive RKO cell line (Fig. 1c), indicating the importance of receptor-mediated signals in GM-CSF-induced EMT program. It was worthy to note that, given that RKO cell line was lack of E-cadherin^28^, we detected another epithelial marker Keratin 18 and showed no alteration of this marker expression following GM-CSF treatment (Fig. 1c). To further address this issue, we established the cell clones in SW480 cell lines transduced with GM-CSF-expressing construct (Fig. S2a). Compared with empty vector-transduced cell clones, GM-CSF–overexpressing clones exhibited EMT phenotypes (Fig. S2b and c), similar to those with stimulation of exogenous GM-CSF. Overall, these data suggest an EMT-inducing effect of GM-CSF exerted directly on colon cancer cells. In addition, we found that compared with its counterpart SW480 cell line, SW620 exhibited mesenchymal phenotype (Fig. S3a) and constitutively produced much more GM-CSF than SW480 (Fig. S3b). Intriguingly, blockade of GM-CSF bioactivity using neutralizing monoclonal antibodies rendered MET of SW620, including increased E-cadherin and decreased vimentin (Fig. S3c), indicating that GM-CSF may be a key factor for EMT program and maintenance.

### GM-CSF stimulation enhances the ability of motility in colon cancer cells

**Figure 2:**
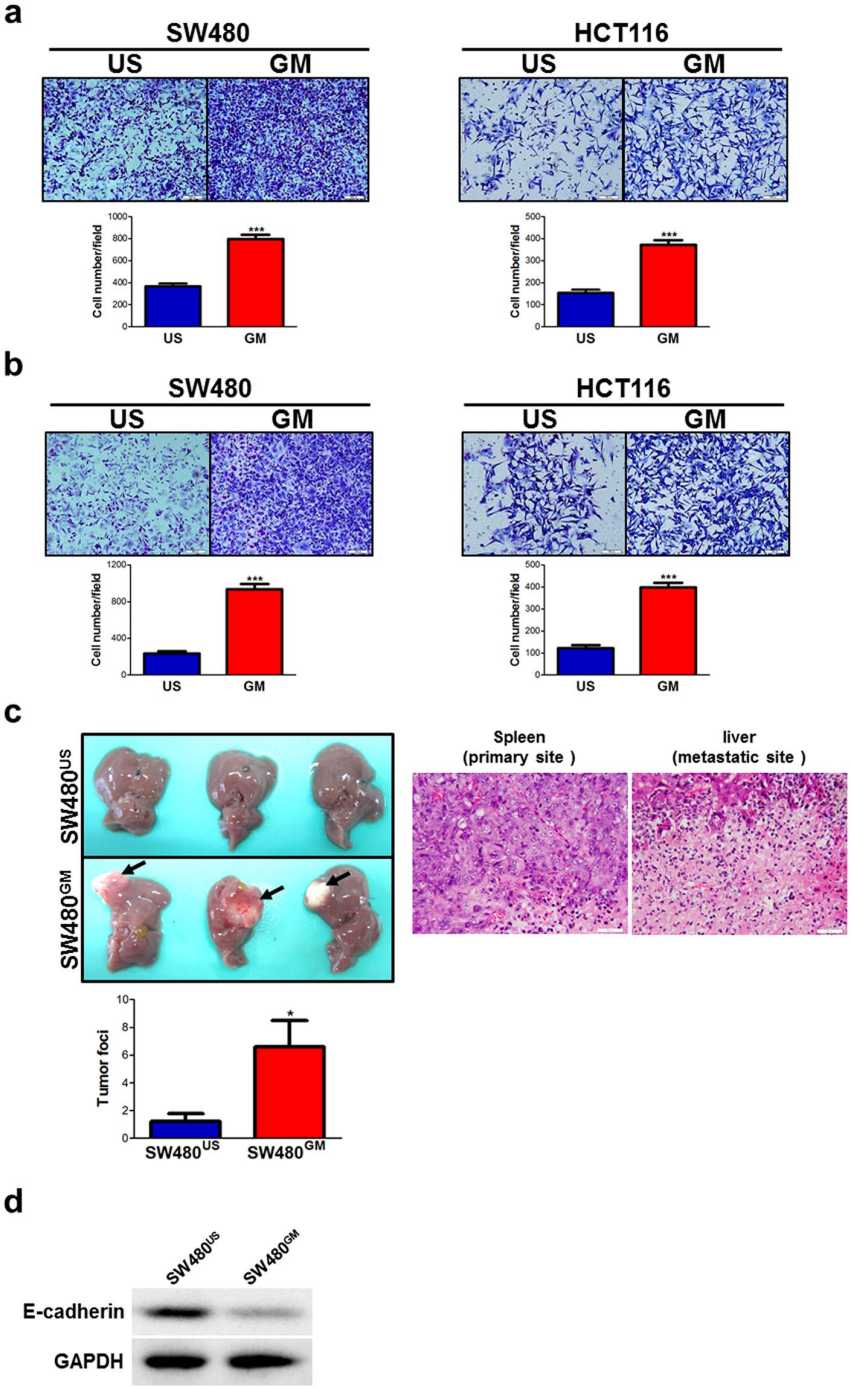
GM-CSF-exposed colon cancer cells exhibit augmented motility *in vitro* and *in vivo*. (**a**,**b**) SW480 and HCT116 colon cancer cell lines were treated with GM-CSF (25 ng/ml) for three weeks. The ability of migration (**a**) and invasion (**b**) was examined by transwell experiments. Upper: representative images were shown. Bottom: the data were pooled from three experiments. (**c**) SW480 cell lines were treated with GM-CSF as described above and collected as well as transfused to nude mice in a model of colorectal cancer liver metastasis as described in Materials and methods. Four-to-six weeks later, livers were pooled and tumor foci per mouse were calculated. Left panel: Representative macroscopic images were shown. The arrows denoted metastatic lesions. Right panel: Representative microscopic images of primary (spleen) and metastatic (liver) tumor sites were shown. Scale bar: 50 μm. The data were pooled from three experiments. (**d**) Tumors in spleen of recipients were collected and E-cadherin expression was detected by immunoblotting. Cropped blots were shown. *P < 0.05; **P < 0.01; ***P < 0.001 vs untreated controls.

Next, we evaluated the migration and invasion of colon cancer cells with or without stimulation of GM-CSF in transwell experiments. Chronic exposure to GM-CSF, indeed, significantly augmented the capacity of migration and invasion *in vitro* (Fig. 2a and b). This effect was also confirmed by wound-healing assays (Fig. S4). Of note, this did not arise from the increase in the cell number following GM-CSF stimulation, as chronic exposure to GM-CSF did not render robust proliferation of cancer cells (Fig. S5). Furthermore, in a mouse model of colorectal cancer liver metastasis, these GM-CSF-educated colon cancer cells displayed more movement to target organ (Fig. 2c). Accordingly, these metastatic cancer cells exhibited mesenchymal phenotype (Fig. S6). Similar phenotype was observed in GM-CSF-overexpressing HCT116 cell line (Fig. S7a and b). Of note, upon inoculation of cancer cells into spleen, exogenous GM-CSF was removed in four-to-six week intervals. E-cadherin expression in GM-CSF-educated cancer cells isolated from splenic inoculation site, however, was much lower than that in untreated counterparts (Fig. 2d), indicating that colon cancer cells at sites of injection still harbored the mesenchymal phenotype. Thus, we can make a conclusion that GM-CSF stimulation sufficiently enhances the capacity of motility of colon cancer cells *in vitro* and dissemination *in vivo*.

### MAPK/ERK-ZEB1 signals are required for GM-CSF-induced EMT program

**Figure 3:**
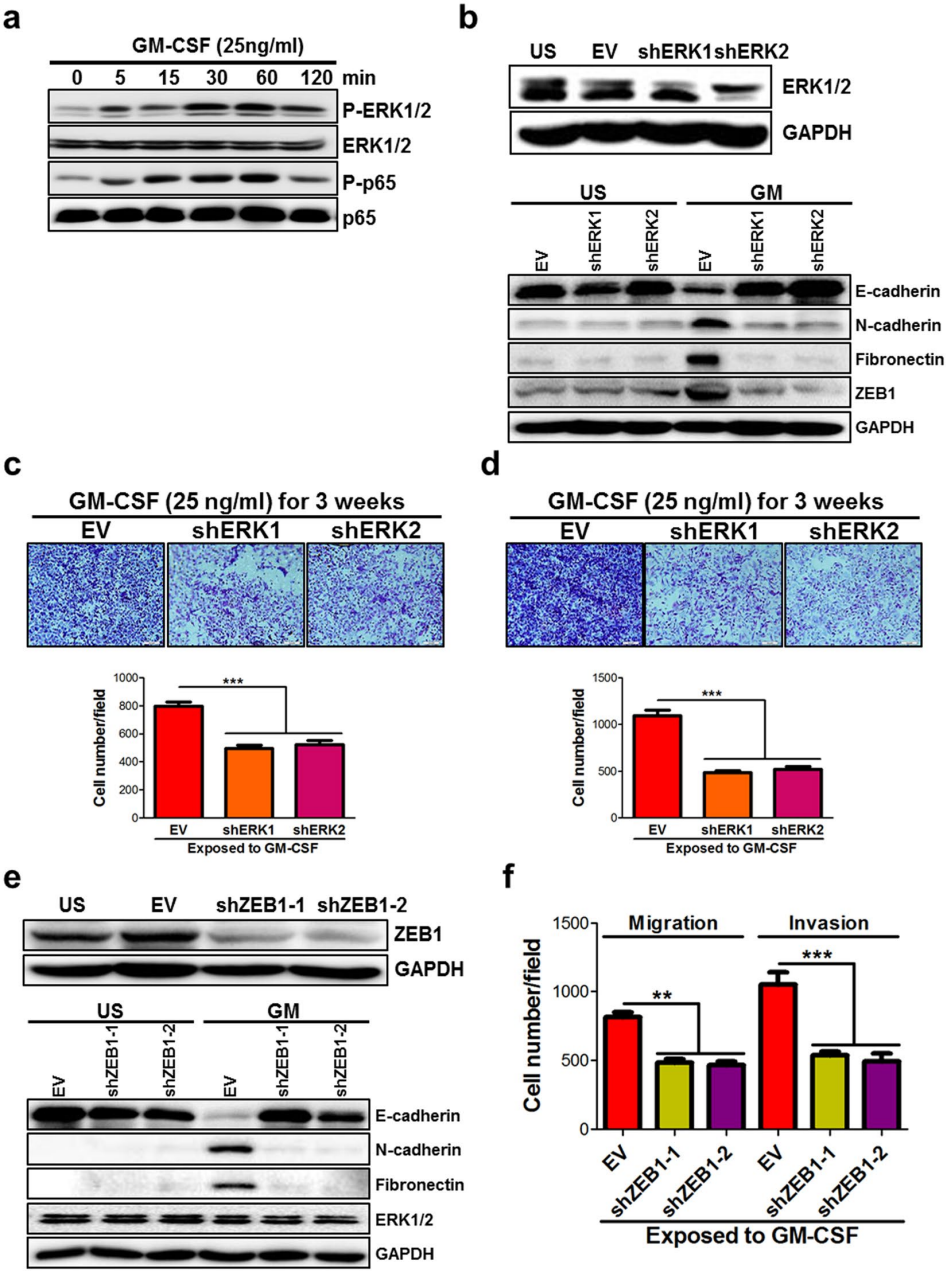
GM-CSF induces EMT program through MAPK/ERK-ZEB1 signaling pathway. (**a**) SW480 cell line was stimulated with GM-CSF (25 ng/ml) for 5–120 min. At the indicated timepoints, the proteins were extracted and the phosphorylation of ERK1/2 and NF-κB p65 subunit was detected by immunoblotting. Representative data of cropped blots from three independent experiments were shown. (**b**) ERK1 and 2 in SW480 cell line were knockdown by RNA interference and cells were stimulated with GM-CSF (25 ng/ml) for three weeks. The expression of E-cadherin, N-cadherin, fibronectin and ZEB1 was examined by immunoblotting. Cropped blots were shown. (**c**,**d**) ERK1 and 2-knockdown SW480 cell line was treated with GM-CSF as described above. The ability of migration (**c**) and invasion (**d**) was detected by transwell experiments. (**e**) ZEB1 in SW480 cell line was knockdown by RNA interference and cells were stimulated with GM-CSF as described above. The expression of E-cadherin, N-cadherin, fibronectin and ERK1/2 was examined by immunoblotting. Cropped blots were shown. (**f**) ZEB1-knockdown SW480 cell line was treated with GM-CSF as described above. The ability of migration and invasion was detected by transwell experiments. Upper: representative images were shown. Bottom: the data were pooled from two experiments. EV: empty vector. **P <  < 0.01; ***P < 0.001 vs EV controls.

It is well-known that the binding of GM-CSF to its receptor activates at least three signaling pathways: JAK/STAT, mitogen-activated protein kinase (MAPK), and PI3K^29^. Thus, we detect the activation of these pathways in colon cancer cell lines upon GM-CSF stimulation. The results showed that the activation of STAT3/5 was not seen in GM-CSF-pulsed SW480 cells, and the PI3K/AKT signaling also was not disturbed (data not shown). In contrast, the phosphorylation of MAPK/ERK and NF-κB was visible in SW480 cells upon stimulation with GM-CSF (Fig. 3a), which was consistent with our previous study using a murine colon carcinoma cell line^27^. Furthermore, to define the signaling pathways required for GM-CSF-induced EMT program, ERK1 and 2 in SW480 cells were knockdown by RNA interference (Fig. 3b). Deletion of ERK1 or 2 had no significant impact on EMT marker expression in resting cancer cells (Fig. 3b). Conversely, ERK1 or 2 knockdown significantly abrogated EMT program following GM-CSF stimulation (Fig. 3b), including up-regulation of E-cadherin and down-regulation of N-cadherin and fibronectin expression. Moreover, the motility of GM-CSF-educated cancer cells was impaired pronouncedly when ERK1 or 2 was deleted (Fig. 3c and d). These findings indicate that ERK1 or 2 plays a nonredundant role in GM-CSF-induced EMT program.

Intriguingly, ZEB1 expression was dramatically suppressed in ERK1 or 2-knockdown cancer cells with stimulation of GM-CSF (Fig. 3b), suggesting that ZEB1 may represent a key factor to mediate EMT program following GM-CSF stimulation. To address the contribution of ZEB1 to GM-CSF-induced EMT program, ZEB1 was knockdown in SW480 cells (Fig. 3e). As expected, knockdown of ZEB1 did not significantly impact E-cadherin expression in resting SW480 cells, but drastically perturbed GM-CSF-induced EMT program (Fig. 3e). Consistently, the migration and invasion of GM-CSF-educated cancer cells decreased when ZEB1 was knockdown (Fig. 3f). Notably, the amounts of total ERK1/2 protein and their activation were not affected in ZEB1-silenced cancer cells with or without GM-CSF stimulation (Fig. 3e and data not shown). Therefore, MAPK/ERK-ZEB1 signaling pathways are critical for GM-CSF-induced EMT program.

### Chronic stimulation of colon cancer cells with GM-CSF renders chemoresistance

**Figure 4:**
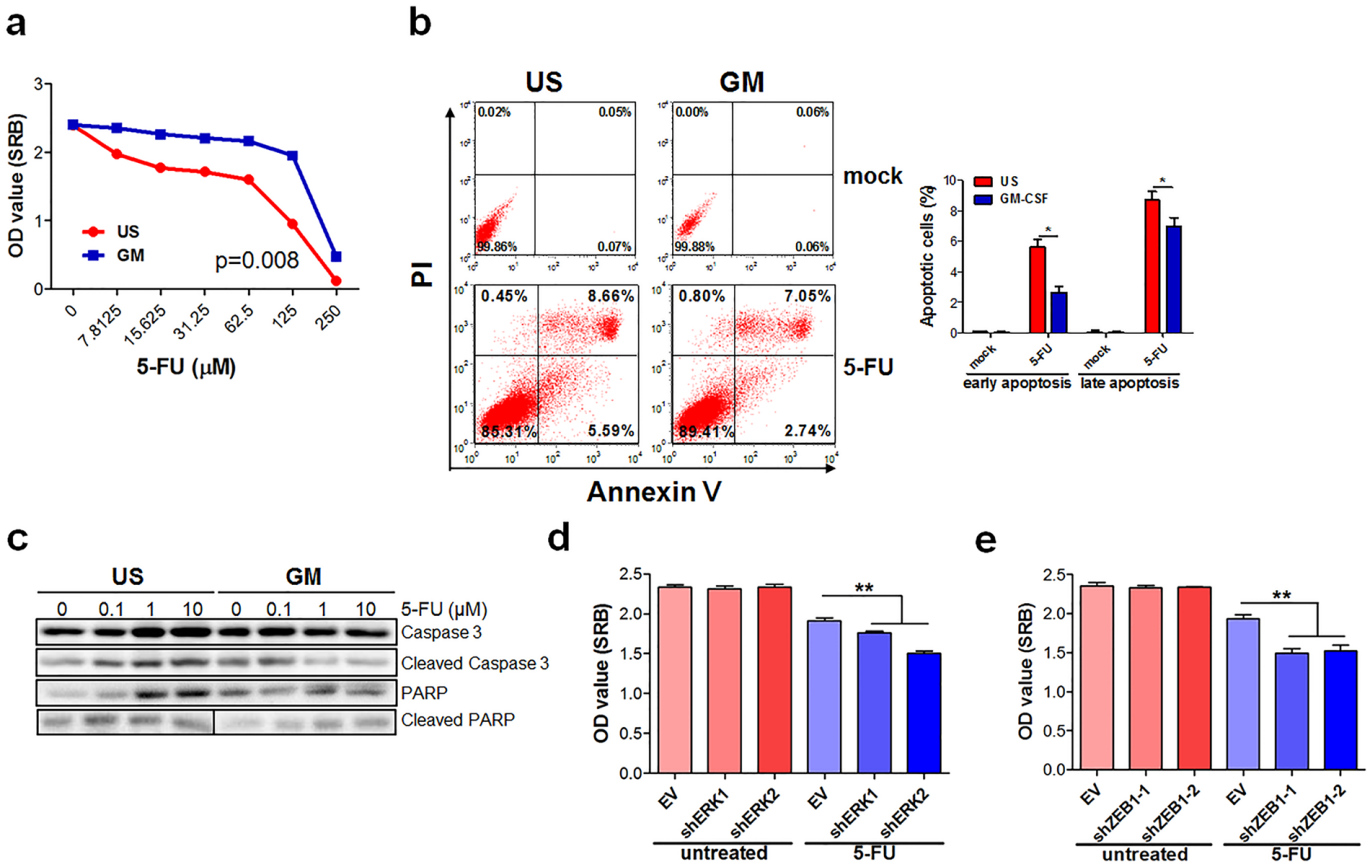
GM-CSF-exposed colon cancer cells exhibit chemoresistance. (**a**,**b**) SW480 cell line was stimulated with GM-CSF (25 ng/ml) for three weeks and then treated with fluorouracil (5-FU) at the titrated concentrations for three days. Cell vitality was detected by SRB assays (**a**) and flow cytometry (**b**). (**c**) SW480 cell line was exposed to GM-CSF as described above and treated with 5-FU at different doses indicated for 24 hours. Total and cleavaged caspase 3 and PARP proteins were examined by immunoblotting. Cropped blots were shown. (**d**,**e**) SW480 cell line with knockdown of ERK1, 2 (**d**) or ZEB1 (**e**) was stimulated with GM-CSF as described above and treated with 5-FU (25 μM) for three days. Cell vitality was detected by SRB assays. The data were pooled from three independent experiments. One-way ANOVA methods were used to determine statistical significance for cell viability test. **P < 0.01; ***P < 0.001 vs untreated or EV controls.

EMT is well-known to intimately correlate with acquisition of drug resistance^30^. To determine whether chronic exposure to GM-CSF led to resistance to cytotoxic compounds, SW480 cells with or without GM-CSF stimulation were treated with three first-line chemotherapeutic agents for colorectal cancer in the clinic. The results showed that, although GM-CSF stimulation did not affect cell viability, GM-CSF-educated cancer cells were resistant obviously to fluorouracil (5-FU), oxiliplatin or irinotecan-mediated cell death, by SRB assays (Fig. 4a and S8a) and Annexin V/PI analysis (Fig. 4b and S8b). This was also reflected by decreased cleavage of caspase 3 and PARP in GM-CSF-stimulated cancer cells, two key proteins which were involved in drug-induced apoptosis (Fig. 4c). Furthermore, knockdown of ERK1/2 or ZEB1 attenuated chemoresistant potentials of GM-CSF-educated cancer cells (Fig. 4d and e).

### High GM-CSF expression in tumor tissues correlates with local metastasis

**Figure 5:**
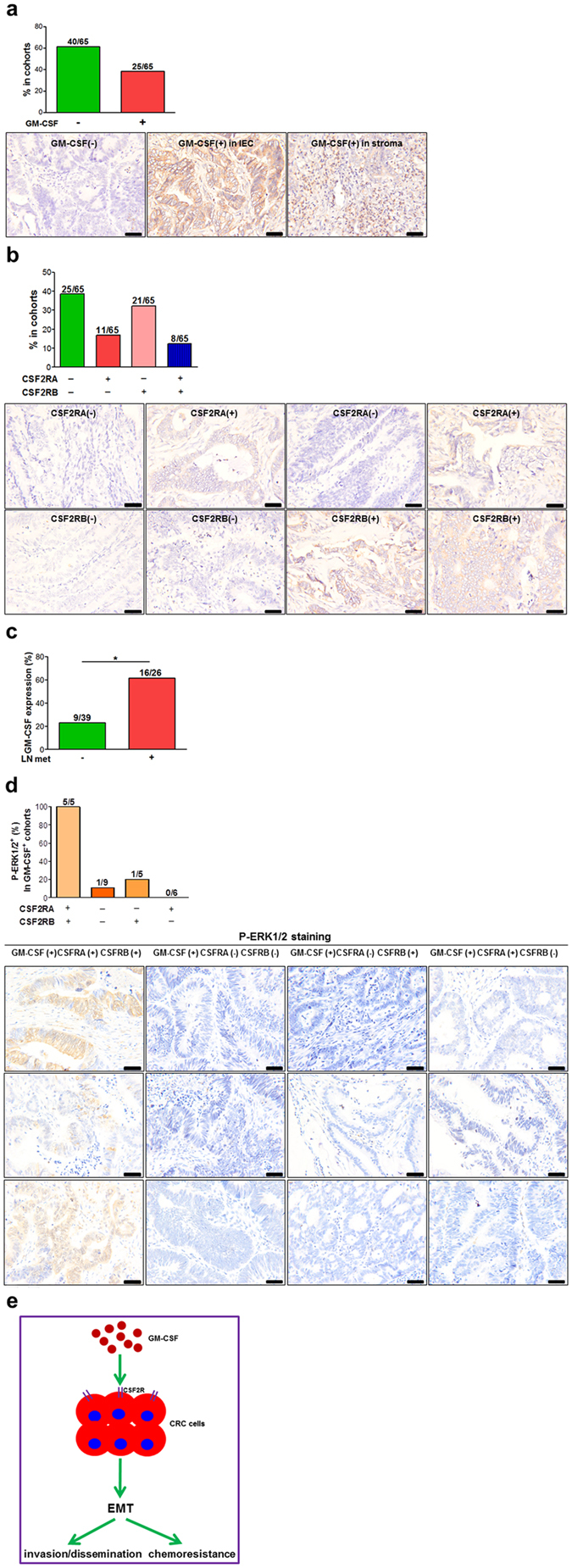
Intimate correlation of GM-CSF expression in CRC specimen with invasion/metastasis. (**a**) GM-CSF expression in tumor tissues of CRC patients was detected by immunohistochemistry. Representative images were shown. Scale bar: 50 μm. The percentages of GM-CSF-positive specimen in the cohorts were calculated. (**b**) The subunit α and β of GM-CSF receptor in tumor specimen were examined by immunohistochemistry. Representative images were shown. Scale bar: 50 μm. The percentages of CRC specimen with GM-CSF receptor-positive in cancer cells in the cohorts were calculated. (**c**) CRC patients were divided into two cohorts: with lymph node (LN) metastasis and without LN metastasis. The percentages of CRC specimen with positive staining of GM-CSF were calculated. LN met: lymph node metastasis. (**d**) The expression of phosphorylated ERK1/2 (P-ERK1/2) in CRC specimen was detected by immunohistochemistry. The percentages of P-ERK1/2-positive specimen in the indicated groups were calculated. Representative images (three cases per group) were shown. Scale bar: 50 μm. (**e**) The outline of our hypothesis as following: GM-CSF in tumor niches drives EMT program in receptor-expressing colon cancer cells and thereby promotes invasion/distant dissemination as well as resistance to chemotherapy. *P < 0.05.

To determine whether our findings are clinically relevant, colon specimens were collected from patients with colorectal cancer. Thirty-eight percentages (25/65) of specimens were positive for GM-CSF staining (Fig. 5a). The expression of GM-CSF in stroma and/or cancer cells was observed, depending on individuals (Fig. 5a). The receptors A and B for GM-CSF were positively stained in cancer cells in 12% (8/65) of specimens (Fig. 5b). Importantly, high GM-CSF expression in cancer samples significantly correlated with lymph node metastasis (Fig. 5c). Furthermore, the receptor-positive cancer samples with high GM-CSF expression frequently harbored activated ERK1 and 2 (Fig. 5d). These data are consistent with the notion that GM-CSF promotes the motility of cancer cells and metastasis by activating MAPK/ERK signaling pathways.

## Discussion

In a previous work we showed that GM-CSF stimulation in colon cancer cell lines could augment the migration and invasion *in vitro* ^27^. Here, we have addressed mechanistic clues underlying these effects, by analyzing EMT-inducing potentials of GM-CSF. Of note, it has been reported that GM-CSF contributes to acquisition of the invasive phenotypes in breast cancer cells by educating infiltrating macrophages in microenvironment^7^. In this study, we provide evidence for the first time that GM-CSF has an effect on the induction of EMT program in receptor-expressing colon cancer cells through activating MAPK/ERK-ZEB1 signaling pathways. These data may provide mechanistic explanation for recent observations that high GM-CSF contents in serum of CRC patients significantly correlated with poor prognosis^3^.

However, the relevance of GM-CSF to CRC progression and prognosis remains controversial. Nebiker *et al*. reported that GM-CSF production by tumor cells was associated with lower T stage and significantly longer survival as well as favorable prognosis in mismatch-repair proficient colorectal cancer^31^. This observation coincides with a previous report showing that 100% of the patients with human colon tumors that overexpressed GM-CSF and its receptors survived at least 5 years after diagnosis^32^. These divergences may be reconciled by the concept that the function of GM-CSF in tumor niche is context-dependent. GM-CSF stimulation alone is potent to drive M1 polarization of macrophage and maturation of dendritic cells, two populations mounting robust anti-tumor immune responses. On the other hand, cancer cell-derived GM-CSF can shape immunosuppressive microenvironment by promoting the generation and accumulation of myeloid suppressor cells and tumor-associated macrophages^7, 9^. The latter indicates that synergistic effects of other soluble factor (s) from cancer cells or stroma may be indispensible for GM-SCF-induced tumor-promoting environment. Besides immunomodulation of tumor-surrounding stromal components, we showed a stimulatory effect of GM-CSF on cancer cells themselves. Clinical analysis also supports the metastasis-promoting role of this cytokine. These data are not in agreement with a previous study that colorectal tumors substantially overexpressed GM-CSF and this cytokine had an immune-independent antitumor effect on receptor-positive cancer cells^32^. It should to be noted that the expression of proinflammatory cytokines such as IL-17A, IL-6, IL-22 was dramatically increased in lesions of colorectal cancer and their high expression significantly correlated with cancer progression and poor prognosis^33–35^. As one member of proinflammatory mediators, it seems plausible that high expression of GM-CSF in tumor is related to more invasive phenotype and poor outcome. Considering a strongly positive correlation of high GM-CSF expression in tumor with distant dissemination and unfavorable prognosis in several types of solid cancers, further investigations of GM-CSF role in CRC development and progression in larger cohorts of CRC samples are warranted.

Mechanistic investigations in this study reveal that MAPK/ERK signaling pathways are required for GM-CSF-induced EMT program and metastasis. Although several signaling pathways have been shown to be activated upon the engagement of GM-CSF with its receptor in hematopoietic cells, MAPK/ERK signals appear to dominantly mediate bioactivity of GM-CSF in epithelial cells, which is consistent with our previous results in a murine colon carcinoma cell line^27^. Furthermore, MAPK/ERK signaling pathways are clearly linked to tumor invasion and metastasis in response to a variety of intracellular and extracellular stimuli in several types of solid cancers including colorectal cancer^22, 36–40^. Of note, we showed that ERK1 and 2 are equivalently important for GM-CSF-induced EMT program, as knockdown of ERK1 or 2 had similar effects on the expression of EMT markers and cell motility. This is not consistent with a recent study showing that ERK2 but not ERK1 is critical for a oncogene placenta-specific 8-driven EMT phenotype in colorectal cancer^41^, suggesting that ERK1 and/or ERK2-mediated EMT lies on upstream clues. We also identified the activation of NF-κB in colon cancer cells upon GM-CSF challenge. The involvement of this key transcriptional factor in GM-CSF-induced EMT need to be addressed in the future through inactivation of NF-κB by ectopic overexpression of IκBα in cancer cells.

As described in previous studies that demonstrated a key role of ZEB1 in mediating EMT and tumor invasiveness in colorectal^42, 43^ and lung cancer^44^, we showed that ZEB1 was also a critical mediator for GM-CSF-driven EMT. ZEB1 expression seems to be regulated by MAPK/ERK signals, because knockdown of ERK1 or 2 decreased ZEB1 expression in GM-CSF-stimulated cancer cells. As previously reported^45, 46^, the activation of MAPK/ERK pathway induced Ets1 and Fra1 expression in cancer cells, and the latter activated ZEB1 promoter and induced its expression, which may provide some clues for the exact regulation of ZEB1 expression by MAPK/ERK signaling pathway in our model. In addition, one of metastasis-promoting function of ZEB1 is reported to enhance MMP secretion^47^, which was increased in GM-CSF-overexpressing HT-29 colon cancer cell lines as described recently^22^. Thus, ZEB1 could regulate colon cancer invasiveness by inducing EMT and MMP production.

It is well established that chronic exposure to a variety of growth factors or cytokines is linked to acquisition of chemoresistance in solid tumors^48^. The role of GM-CSF, however, in this process is unknown. In this paper, we identified GM-CSF as a chemoresistance-inducing factor, at least in colorectal cancer. Given the bioactivity of GM-CSF in other types of tumors, it is plausible that GM-CSF induces chemoresistant phenotype in receptor-expressing cancer cells. Furthermore, we showed that chemoresistance induced by chronic stimulation with GM-CSF was tightly associated with EMT, which is consistent with the notion that EMT contributes to drug resistance, which is validated in many types of tumors. Accordingly, since knockdown of ERK1/2 or ZEB1 led to the failure of GM-CSF-driven EMT, it is reasonable that inhibition of MAPK/ERK-ZEB1 signals also abrogates acquisition of chemoresistance. Notably, the contribution of MAPK/ERK-ZEB1 signaling pathway to multidrug resistance has been reported in numerous types of cancer, including breast cancer^49^, hepatocellular carcinoma^50^, and glioblastoma^51^. Therefore, inhibition of MAPK/ERK signals using small-molecular compounds may be favorable to reverse chemoresistance in certain settings. The details in the regulation of chemoresistant traits in cancer cells by MAPK/ERK-ZEB1 pathways need to be further addressed in the future, although GM-CSF stimulation greatly suppresses chemo-induced active cleavage of caspase and PARP.

In conclusion, in this study we demonstrate that GM-CSF is an EMT-inducing factor by binding its receptor on the surface of cancer cells through activating MAPK/ERK-ZEB1 signaling pathway (Fig. 5e). Of note, our data showed that EMT occurred on day 7 after GM-CSF stimulation, instead of day 3, indicating that products of early response genes within 3 days are not sufficient to induce EMT. Therefore, it may be true that early signaling pathways activated by GM-CSF involving MAPK/ERK are important to induce other EMT-inducing factors such IL-6, IL-1β and VEGF, whose consistent productions act in concert to induce EMT features. In support of this assumption, we showed previously that GM-CSF potently induced other proinflammatory cytokine expression in a murine colon cancer cell line^27^. Thus, further work is warranted to determine whether any one of them is responsible for mediating GM-CSF effect on EMT by adding neutralizing antibodies to some of these factors during the culture.

Currently, functional contribution of GM-CSF to cancer progression and metastasis is controversial, which may be due to dual effects of GM-CSF on immune cells and non-immune cells. Considering that recombinant GM-CSF are routinely used to correct neutropenia subsequent to chemotherapy and radiation and that adjuvant GM-CSF treatment has been suggested to occasionally enable tumor growth, detection of GM-CSF receptor expression in cancer cells is warranted in the future prior to GM-CSF treatment, to better define eligile patient’s cohorts.

## Methods

### Tissue samples

Primary CRC tissue samples were obtained from 65 patients undergoing surgical resection at the Department of Surgery, Zhengzhou Yihe Hospital from February 2010 to June 2011. Sections were reviewed by two experienced pathologists to verify the histologic assessment. All the specimens were adenocarcinoma. Prior informed consent was obtained and the study protocol was approved by the Ethics Committee of Zhengzhou Yihe Hospital. All experiments were performed in accordance with relevant guidelines and regulations.

### Cell culture and drug treatment

Human colon adenocarcinoma cell lines (SW480, SW620, HT29, HCT116, DLD-1, RKO) and human acute monocytic leukemia cell line THP-1 were purchased from American Type Culture Collection and maintained in appropriate medium according to product specification, supplemented with 10% fetal bovine serum, 100 units/ml penicillin and 100 mg/ml streptomycin. Cells were seeded in 6-well culture plates (1 × 10^6^/ml/well) and stimulated with recombinant human GM-CSF (Peprotech) at 1–100 ng/ml for 1–3 weeks. To blocking GM-CSF activity, neutralizing anti-GM-CSF monoclonal antibody or isotype IgG (1 μg/ml, Biolegend) was added into the culture and maintained for one week. Then cells were collected and centrifuged at 1500 rpm for 5 minutes and resuspended in serum-free medium. In some settings, cells were treated with fluorouracil (5-FU), oxiliplatin and irinotecan at indicated concentration for 72 hours, which were purchased from Selleckchem. After incubation, cells were collected for analysis.

### Experimental liver metastasis

The surgical procedure was performed according to splenic injection model as described previously^52^. Briefly, 5- to 6-week-old athymic nude mice (Jackson Laboratory) were anesthetized with ketamine/dexmedetomidine admixture and the spleen was exteriorized through a left lateral flank incision. Tumors were established by intrasplenic injection of 2 × 10^6^ cells suspended in 50 μl of serum-free media using one 27-gauge needle. The injection site of the spleen was pressed with a cotton stick wet in iodine-polividone solution in order to remove extravasated cells and ensure homeostasis. The peritoneum and skin were closed in a single layer using surgical thread. Mice were sacrificed at four-to-six weeks post-injection and the burden of metastatic liver tumors was calculated. Primary (splenic) tumor mass was dissected and the protein was extracted conventionally for immunoblotting analysis. All animal experiments were performed in accordance with international guidelines for the care and use of laboratory animals and approved by the Animal Ethics Committee of the Institute of Basic Medical Sciences.

### Transwell migration and invasion assays

*In vitro* cell migration and invasion assays were performed using transwell chambers with polyethylene terephthalate membrane (24-well inserts, 8.0 μm; Corning). For the migration assay, 2.5 × 10^4^ cells were added to the top chambers. For the invasion assays, 5 × 10^4^ cells were seeded to the top chambers coated with Matrigel (BD Biosciences). Complete medium was added to the bottom wells. After incubation for 36 hours, the noninvasive cells were removed with a cotton swab. The cells that had migrated through the membrane and adhered to the lower surface of the membrane were fixed with methanol for 10 minutes and stained with crystal violet solution (0.1%). For quantification, the cells were counted using a microscope from five randomized fields at ×200 magnification.

### Wound-healing assay

Cell migration was also assessed using an *in vitro* scratch wound healing assay. Cells were starved overnight with serum-free RPMI1640 medium and a scratch was made using a sterile 1-mL pipette tip. The cell debris was removed with PBS and cells were cultured with GM-CSF (25 ng/ml) for 24 hours. An inverted microscope was used to observe cell migration.

### Cell proliferation assays

Cell growth was assessed by Sulforhodamine B (SRB) assay. Cells (5 × 10^3^ cells per well in 100 μl medium) were seeded into 96-well plates in triplicate and exposed to recombinant human GMCSF (Peprotech) at titrated concentrations. After incubation for indicated time, 50 μl of 30% trichloroacetic acid was added and incubated for 60 min at 4 °C. After washing and drying the plate, 100 μl of 0.4% SRB was added for 30 min. The plates were rinsed with 0.1% acetic acid and air-dried, after which 100 μl of Tris base (10 mM/L) was added, and the plates were shaken for 5 min. The SRB value was measured at a wavelength of 590 nm.

### Immunoblotting

Samples were homogenized and sonicated in RIPA lysis buffer (Santa Cruz Biotech.), supplemented with protease inhibitors. After centrifugation at 20,000 g for 15 min, 30 μg of the supernatants were separated on 10% SDS-polyacrylamide gel and transferred onto an Immunobilon-P Transfer membrane (Millipore). After being blocked with 5% skim milk, the membrane was incubated with primary antibodies at1:1000 dilution. Rabbit anti-GAPDH antibody was used as an internal control (purchased from Tianjin Sungene Biotech.). ImmunoPure peroxidase-conjugated anti-rabbit IgG were used as secondary antibodies. The blotted membrane was then treated with the Super Signal West Dura Extended Duration Substrate (Pierce) and signals were detected by LAS-3000 mini CCD camera (Fuji Film). Primary antibodies used for immunoblotting were purchased from Cell Signaling.

### Immunohistochemistry

The spleen and liver was removed, fixed in 10% neutral-buffered formalin solution, embedded in paraffin, and cut into tissue sections, which were stained with hematoxylin and eosin. Paraffin-embedded sections were deparaffinized and immersed in 80 °C water bath in 10 mM sodium citrate buffer with 0.1% Tween 20 overnight for antigen unmasking. Slides were incubated with primary antibody against CSF2RA (1:100, Biolegend), CSF2RB (1:80, Lifespan Bioscience), GM-CSF (1:150, Novus Biologicals), P-ERK1/2 (1:400, Cell Signaling), E-cadherin (1:100, Cell Signaling) or Fibronectin (1:100, Cell Signaling) in PBS containing 1% BSA and 10% goat serum. Biotinylated secondary antibodies (Dako) were added and incubated at room temperature for 1 hour. Streptavidin-HRP (BD Pharmingen) was added, and after 40 min the sections were stained with DAB substrate and counterstained with hematoxylin.

### Immunofluorescence

Cells were seeded onto glass coverslips in 24-well plates, washed with PBS, fixed in 4% formaldehyde solution and permeabilized with 0.2% Triton X-100/PBS. Cells were blocked with 2% BSA in PBS for 30 min. Coverslips were incubated with primary antibodies for 1 hour, followed by incubation with phycoerythrin (PE)-conjugated secondary antibodies for 1 hour, and then stained with DAPI. Finally, coverslips were observed under a fluorescence microscope.

### Generation of stable knockdown or knockin cell line

ShRNA plasmids targeting ERK1/2 and non-targeting shRNA control plasmid were kindly provided by Robert J. coffey (Department of Medicine, Vanderbilt University Medical Center)^41^. ShRNA plasmid targeting ZEB1 and human GM-CSF expression plasmid were purchased from Santa Cruz Biotech. SW480 or HCT116 colon cancer cell lines (1 × 10^7^/ml/well) were seeded in 6-well culture plates and transfected with plasmids indicated above using Lipofectamine 2000 (Invitrogen) according to the manufacturer’s protocol. Stable cell lines were selected with 2 μg/ml puromycin.

### Apoptosis asaays

Apoptosis was quantified using fluorescein isothiocyanate (FITC)-Annexin V and propidium iodide (PI), according to the manufacturer’s protocol (ApoDETECT FITC-Annexin V Kit; Zymed Laboratories Inc.). Cells were stained with 5 μl of FITC-Annexin V diluted 1:10 in buffer, and 2.5 μl of PI. After incubation for 15 minutes, cells were analyzed by flow cytometry with standard FACScan equipment (Becton Dickinson Co.).

### ELISA for GM-CSF

GM-CSF levels in the supernatants were measured using ELISA kits (eBiosciences) according to the manufacturer’s protocol.

### Quantitative RT-PCR

RNA was extracted and reverse-transcribed into cDNA using the SuperScript III First Strand cDNA synthesis system (Invitrogen). cDNA was synthesized from 0.5 μg RNA using random hexamer primers and SuperScript III (Invitrogen). Real-time RT-PCR was performed on a Bio-Rad iCycler to quantify mRNA levels. The primers for real-time were listed in Supplementary Table 2. All reactions were performed in triplicate. The data were analyzed using Q-Gene software and expressed as fold change mean normalized expression (MNE) from control value. MNE is directly proportional to the amount of RNA of the target gene relative to the amount of RNA of the reference gene, GAPDH.

### Statistics

Data represent means of multiple determinations and were analyzed using the GraphPad Prism statistical PC program (GraphPad Software, San Diego, California, USA) by means of paired *t* test. One-way analysis of variance (ANOVA test) was used to define differences in cell viability in cultures treated with various concentrations of cytotoxic agents. A value of *P* < 0.05 was considered statistically significant.

## Electronic supplementary material

Supplementary Information
