# Supplementary material for: An epithelial-to-mesenchymal transition-inducing potential of granulocyte macrophage colony-stimulating factor in colon cancer
Source: Sci Rep. 2017 Aug 15;7:8265. doi: 10.1038/s41598-017-08047-1 (PMC5557751; doi:10.1038/s41598-017-08047-1)
Supplement: Supplementary file 1 — Supplementary Information [file 41598_2017_8047_MOESM1_ESM.pdf]

An epithelial-to-mesenchymal transition-inducing potential of granulocyte macrophage colony-stimulating factor in colon cancer

Yaqiong Chen, Zhi Zhao, Yu Chen, Zhonglin Lv, Xin Ding, Renxi Wang, He Xiao, Chunmei Hou, Beifen Shen, Jiannan Feng, Renfeng Guo, Yan Li, Hui Peng, Gencheng Han and Guojiang Chen

Supplementary Tables

Table S1. Summary of clinical features of CRC patients.

Table S2. Primer sequences for quantitative RT-PCR analysis.

Clinical characteristics of the 65 colorectal cancer patients

Clinicopathological features	No. of patients	P value*
Age, year (median, range)	61 (36-82)	ND
Gender		0.56
Male	35	
Female	30	
Tumor location		0.12
Left-sided	54	
Right-sided	11	
Histological grade (WHO)		0.07
G1	4	
G2	31	
G3	30	
pT status		0.09
1	0	
2	14	
3	11	
4	40	
pN status		0.24
0	39	
1	26	
pM status	ND	
Clinical stage		0.18
Ⅰ	12	
Ⅱ	26	
Ⅲ	27	
Ⅳ	ND	

ND: not determined.
*by the student's *t* test or the Mann-Whitney U test.

Primers used for RT-PCR analysis of gene expression.

gene	Forward 5'-3'	Reverse 5'-3'
CSF2RA	GGCACGAGGCGAGAGAAG	CACCCTCTGGGTCTCAGGTA
CSF2RB	CTGTGAAATGGGTCTGGCCT	GGGGGAAAAACACTCCACCA
GM-CSF	GGGAGCATGTGAATGCCATC	GGCTCCTGGAGGTCAAACAT
E-cadherin	TGAAGGTGACAGAGCCTCTGGA	TGGGTGAATTCGGGCTTGTT
N-cadherin	GCGCGTGAAGGTTTGCCAGTG	CCGGCGTTTCATCCCATACCACA[illegible]
Fibronectin	AGGACGGACATCTTTGGTGC	TGTGGTTGTTGTATAGGAAGGG
Vimentin	CCTTGAACGCAAAGTGGAATC	GACATGCTGTTCCTGAATCTGAG
ZEB1	AAGTGGCGGTAGATGGTA	TGTTGTATGGGTGAAGCA
ZEB2	CCAGCGGAAACAAGGATTTCA	GTCAAGTCATCTAGGCCCGACA
SNAIL	CCTCCCTGTCAGATGAGGAC	CCAGGCTGAGGTATTCCTTG
SLUG	CATGCCTGTCATACCACAAC	GGTGTCAGATGGAGGAGGG
TWIST1	GGACAGAGATTCCCAGACGG	GGCTGATTGGCACGACCT
TWIST2	TCGAGAGGCAGCCCAAGCGCT	CTAGTGGGAGGCGGA
GAPDH	AACGGATTTGGTCGTATTG	GTACTCCAGAAGACCAGAGG

Supplementary Figure legends

Figure S1. GM-CSF induces EMT phenotype in colon cancer cells in dose and time-dependent fashion. (a) The expression of GMCSF receptors in colon cancer cell lines was detected by quantitative RT-PCR. (b) HT29 colon cancer cell line was stimulated with GM-CSF (25 ng/ml) for three weeks respectively. The expression of epithelial and mesenchymal markers as well as EMT-related transcriptional factors indicated were examined by immunoblotting. Representative data of cropped blots from three independent experiments were shown. (c) SW480 cell line was stimulated with GM-CSF at the indicated concentrations for three weeks or at the dose of 25 ng/ml for one-to-three weeks respectively. The expression of E-cadherin and N-cadherin was examined by immunoblotting. Representative data from three independent experiments were shown.

Figure S2. Ectopic expression of GM-CSF drives EMT program in colon cancer cells. SW480 cell line was stably transfected with a plasmid encoding human GM-CSF. (a) SW480 cell line with transfection of GM-CSF (GM) or empty vector (EV) as well as parental cell line were cultured in serum-free RPMI1640 medium for 24 hours. The levels of GM-CSF in the medium were detected by ELISA. (b,c) The expression of EMT-related markers and transcriptional factors in GM-CSF-overexpressing cancer cells was examined by immunoblotting (b) and quantitative RT-PCR (c). (d) The ability of migration and invasion of GM-CSF-overexpressing cancer cells was determined by

transwell experiments. Representative data from three independent experiments were shown. *, *P*<0.05; **, *P*<0.01; ***, *P*<0.001 vs EV controls.

Figure S3. Constitutive secretion of GM-CSF is required for maintaining mesenchymal phenotype in colon cancer cells. (a) The protein in SW480 and SW620 cell lines was extracted and the expression of E-cadherin, N-cadherin and vimentin was detected by Western blotting. (b) SW480 and SW620 cell lines were cultured in 24-well plate ($1x10^5$/well) in serum-free RPMI1640 medium for 24 hours. The supernatants were collected and GM-CSF contents were determined by ELISA. (c) Neutralizing anti-GM-CSF monoclonal antibody (1 μg/ml) was added into the culture of SW620 cell line. One week later, cells were pooled and the expression of E-cadherin and vimentin was detected by Western blotting. The data were pooled from three independent experiments. ***, *P*<0.001 vs SW480 cell lines.

Figure S4. GM-CSF stimulation enhances motility of colon cancer cells. HCT116 and SW480 cell lines were stimulated with GM-CSF (25 ng/ml) for three weeks. The ability of migration was determined by wound-healing assay. Microphotographs of the scratches were obtained at 24 hours post-wounding. Scale bar: 100 μm. Representative data from two independent experiments were shown.

Figure S5. Chronic exposure of colon cancer cells to GM-CSF does not affect cell proliferation. SW480 and HCT116 colon cancer cell lines were stimulated with GM-

CSF (25 ng/ml) for 7-21 days respectively. Cell proliferation was determined by SRB assays. Representative data from two independent experiments were shown.

Figure S6. Colon cancer cells at metastatic sites display mesenchymal phenotype. Colorectal cancer liver metastasis model was established as described in Materials and methods. Tumor nodes in spleen and liver were dissected and E-cadherin as well as Fibronectin were detected by immunohistochemistry. Representative data from three independent experiments were shown. Scale bar: 50 μm.

Figure S7. GM-CSF-overexpressing HCT116 colon cells displays enhanced metastatic capacity. (a) HCT116 cell line was stably transfected with GM-CSF-encoding plasmid (GM) or empty vector (EV) and cultured in serum-free RPMI1640 medium for 24 hours. The levels of GM-CSF in the medium were detected by ELISA. ND: no detected. (b) HCT116 cell line with transfection of GM-CSF-encoding plasmid (GM) or empty vector (EV) was transfused into the spleen of nude mice. Six weeks later, the liver was dissected and tumor foci per mouse were calculated. Representative images from two independent experiments were shown. ***, $P<0.001$ vs EV controls.

Figure S8. Chronic exposure to GM-CSF renders colon cancer cell resistance to drug-induced cytotoxicity. (a,b) SW480 cell line was stimulated with GM-CSF (25 ng/ml) for three weeks and treated with oxaliplatin and irinotecan at indicated concentrations respectively. Cell vitality was determined by SRB assays (a) and flow

cytometry (b). The data were pooled from three independent experiments. One-way ANOVA methods were used to determine statistical significance for cell viability test. *, P<0.05; **, P<0.01 vs untreated controls.

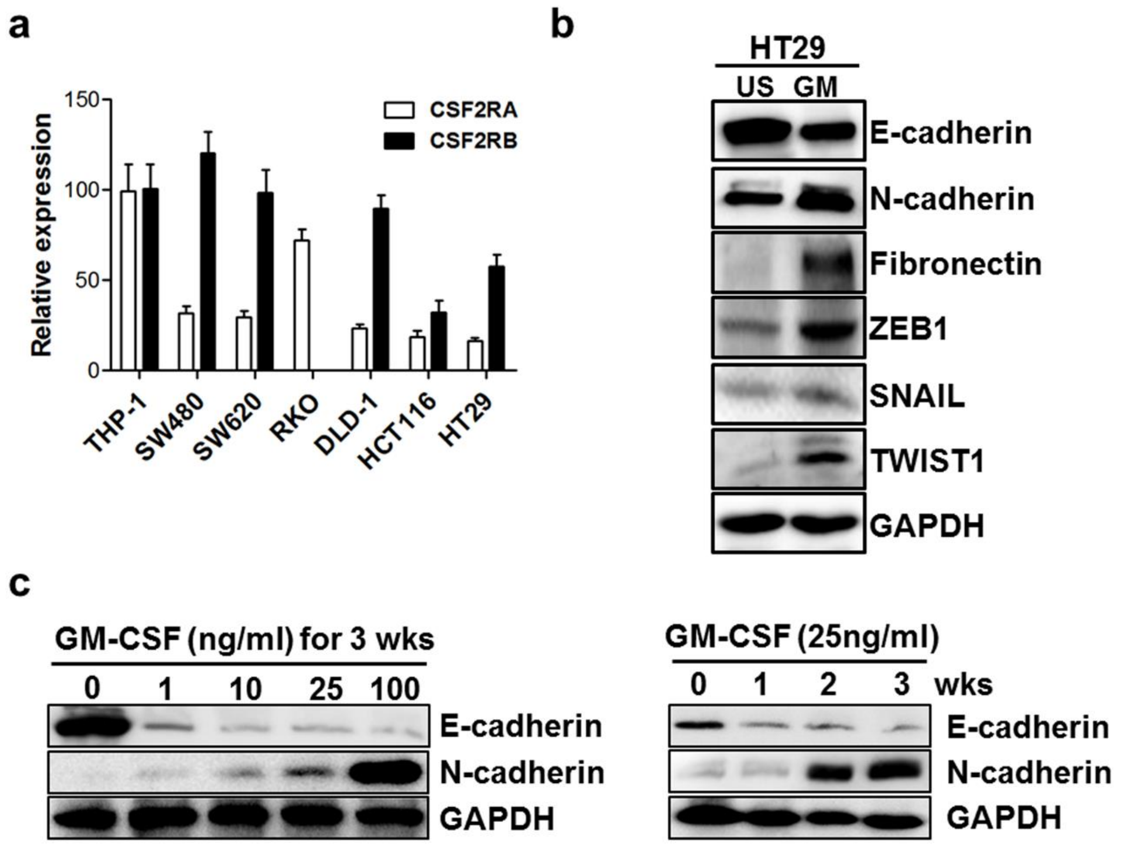
a
Relative expression
150
100
50
0
CSF2RA
CSF2RB
THP-1
SW480
SW620
RKO
DLD-1
HCT116
HT29
b
HT29
US GM
E-cadherin
N-cadherin
Fibronectin
ZEB1
SNAIL
TWIST1
GAPDH
c
GM-CSF (ng/ml) for 3 wks
0 1 10 25 100
E-cadherin
N-cadherin
GAPDH
GM-CSF (25ng/ml)
0 1 2 3 wks
E-cadherin
N-cadherin
GAPDH

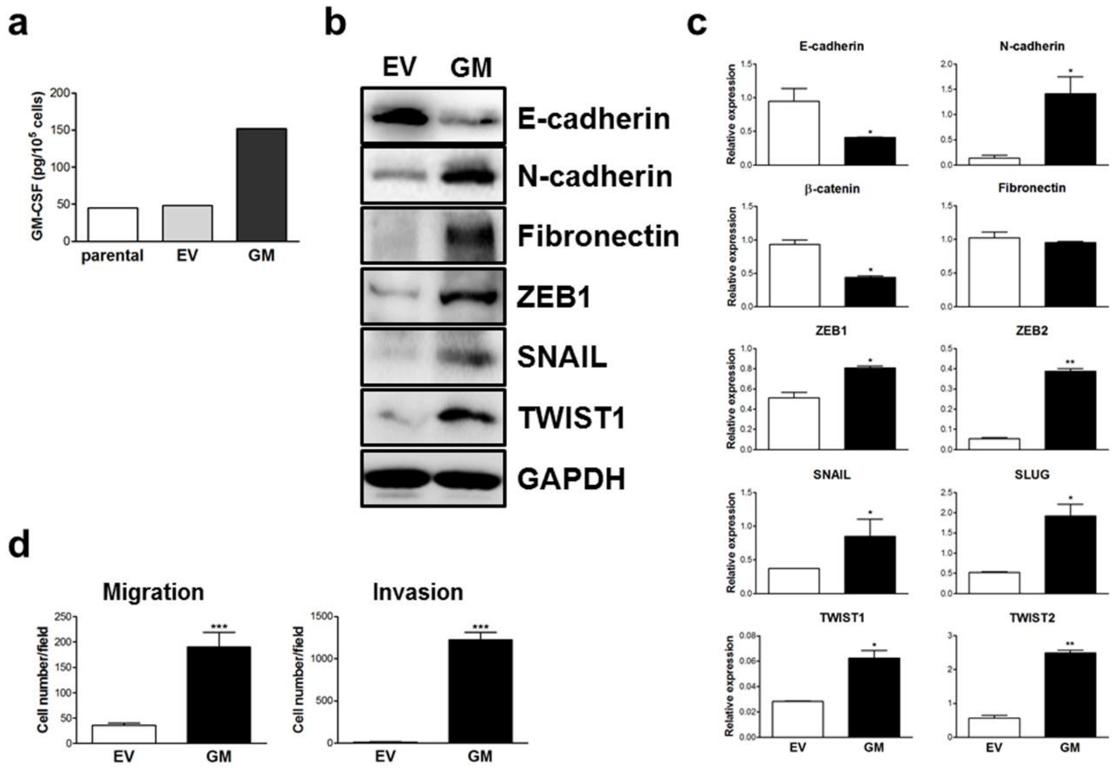
a
GM-CSF (pg/10^5 cells)
parental
EV
GM
b
EV
GM
E-cadherin
N-cadherin
Fibronectin
ZEB1
SNAIL
TWIST1
GAPDH
c
E-cadherin
N-cadherin
β-catenin
Fibronectin
ZEB1
ZEB2
SNAIL
SLUG
TWIST1
TWIST2
Relative expression
d
Migration
Invasion
Cell number/field

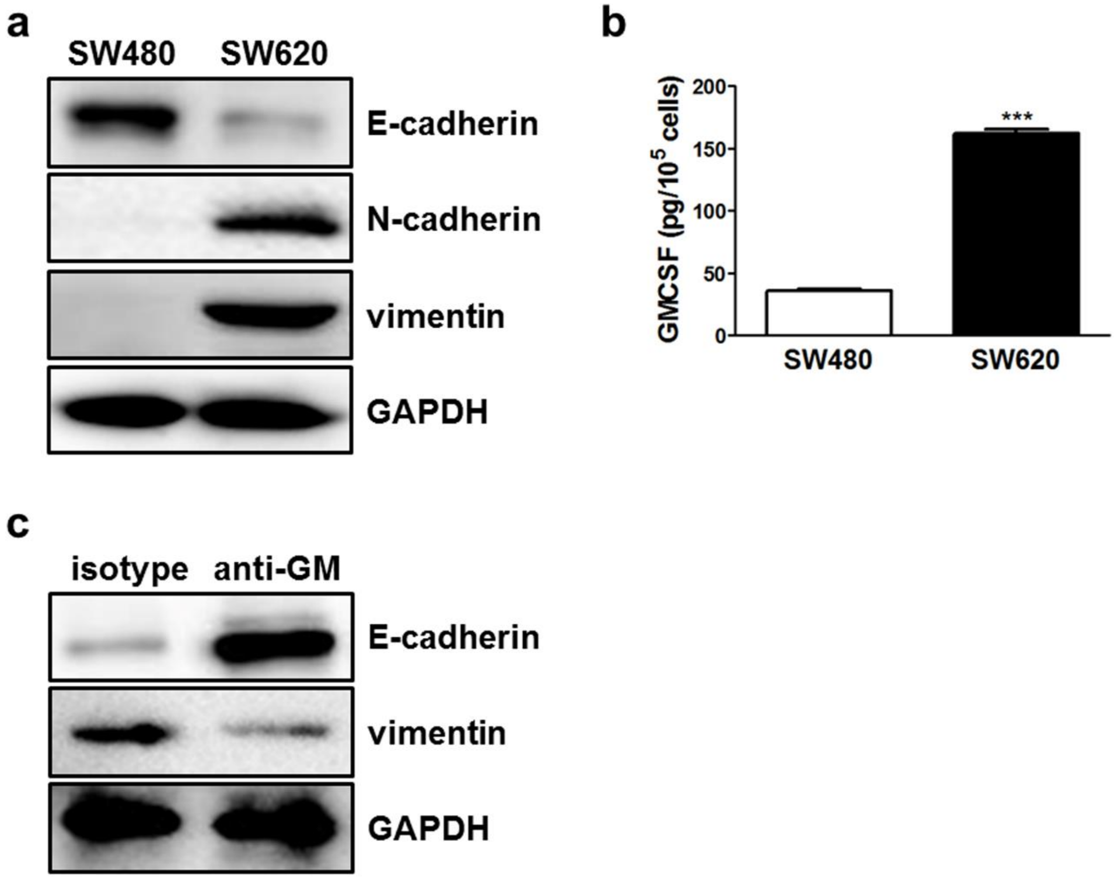
a
SW480
SW620
E-cadherin
N-cadherin
vimentin
GAPDH
b
GMCSF (pg/10^5 cells)
200
150
100
50
0
***
SW480
SW620
c
isotype
anti-GM
E-cadherin
vimentin
GAPDH

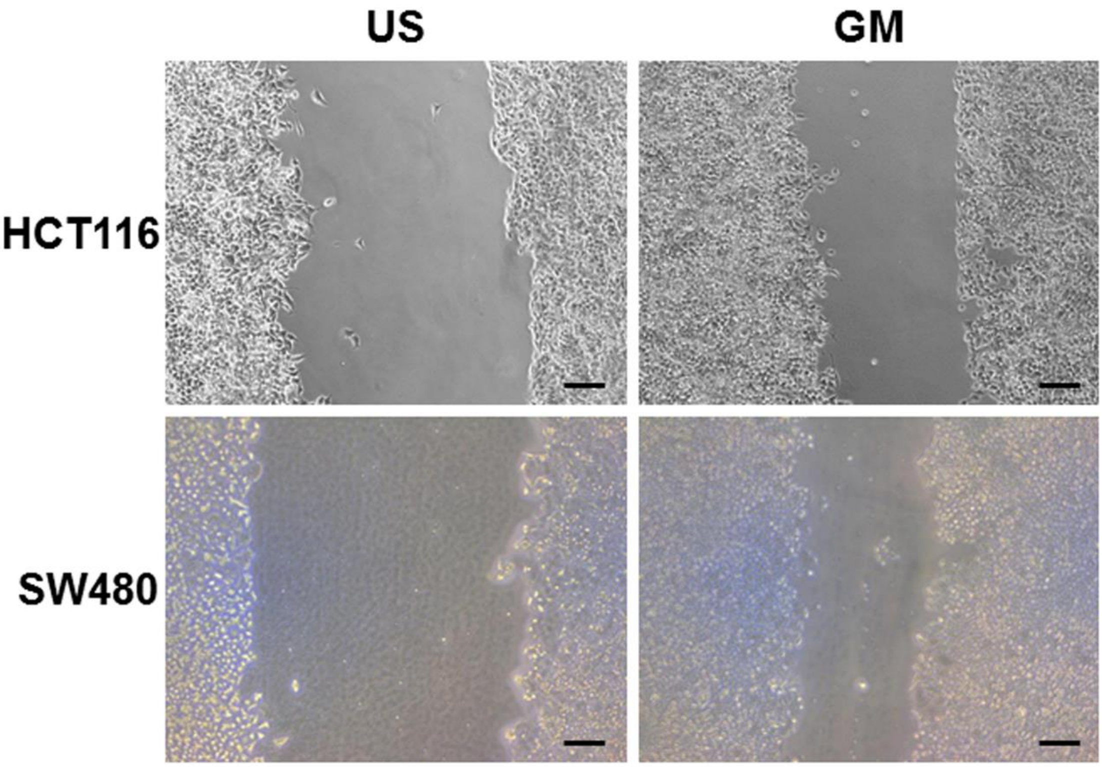
US
GM
HCT116
SW480

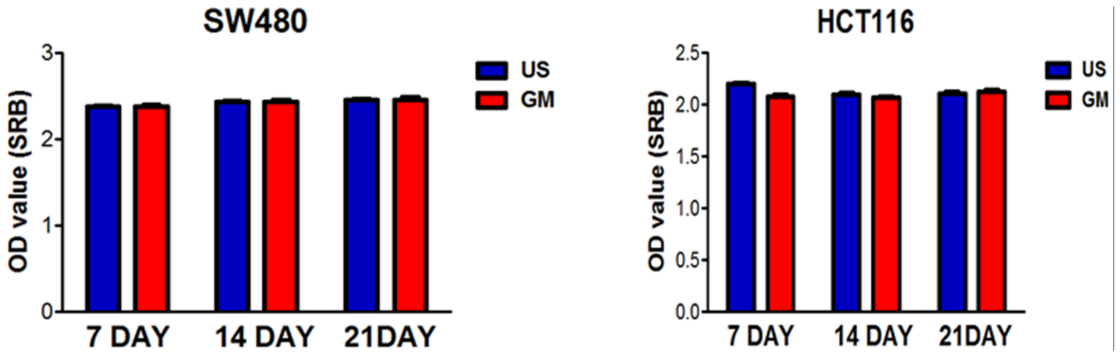
SW480
OD value (SRB)
3
2
1
0
7 DAY
14 DAY
21DAY
US
GM
HCT116
OD value (SRB)
2.5
2.0
1.5
1.0
0.5
0.0
7 DAY
14 DAY
21DAY
US
GM

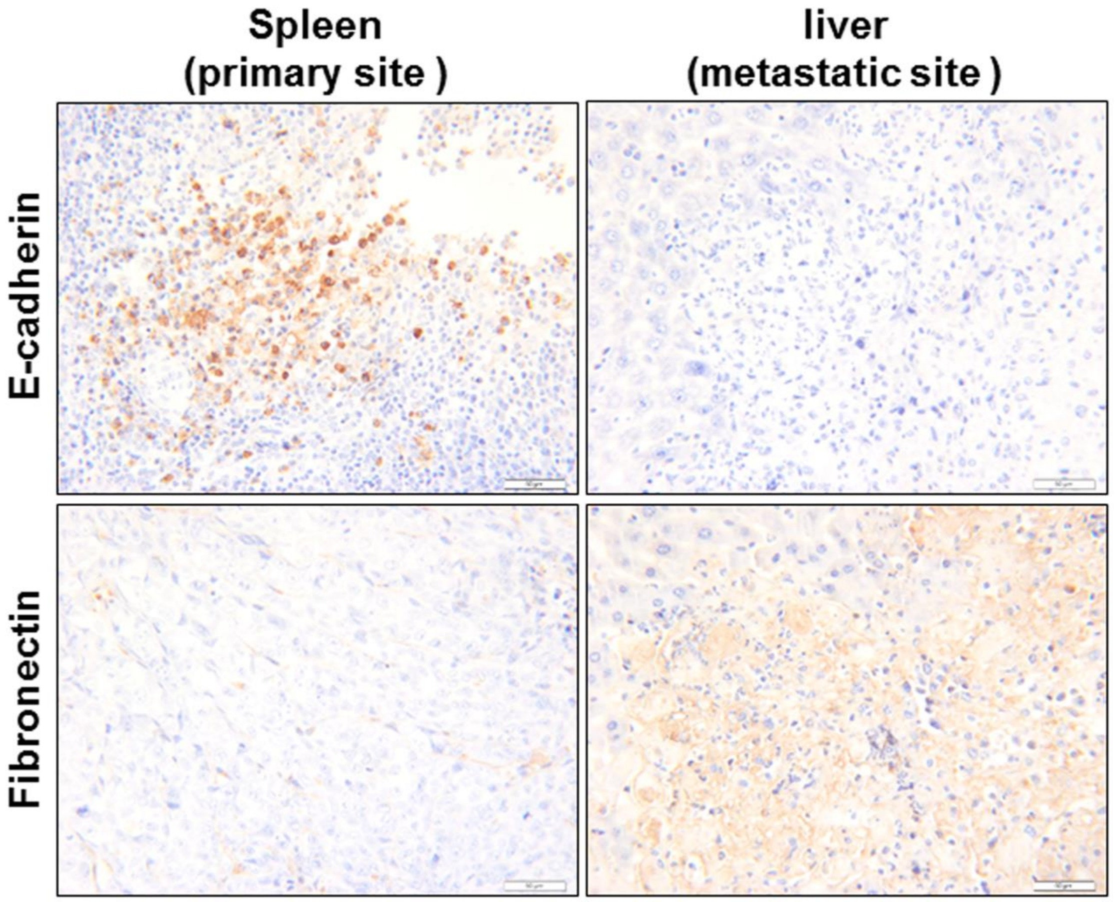

Spleen
(primary site)
liver
(metastatic site)
E-cadherin
Fibronectin

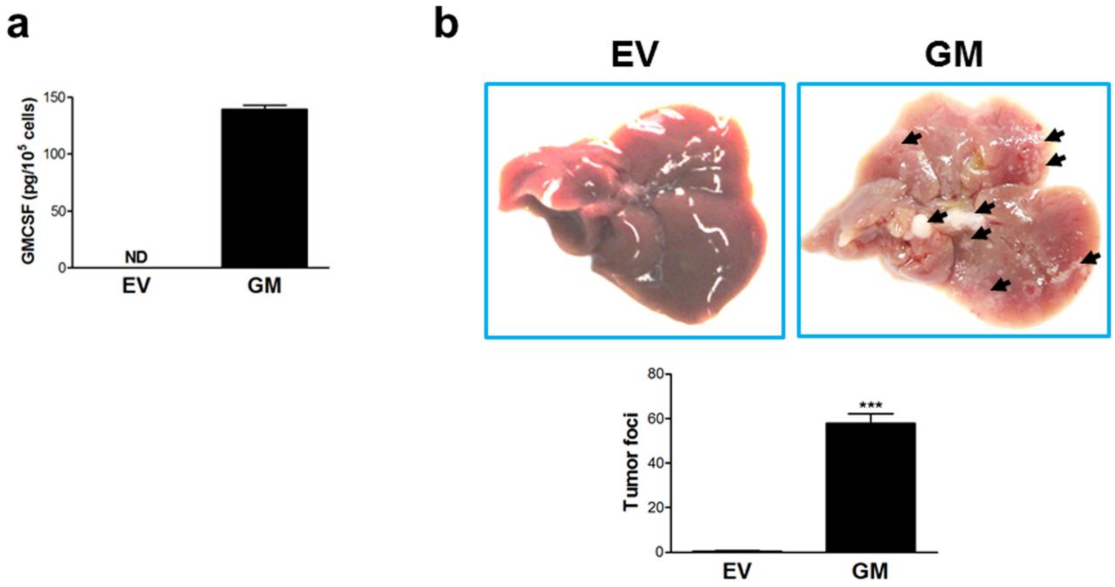
a
b
EV
GM
GMCSF (pg/10^5 cells)
150
100
50
0
ND
EV
GM
Tumor foci
80
60
40
20
0
***
EV
GM

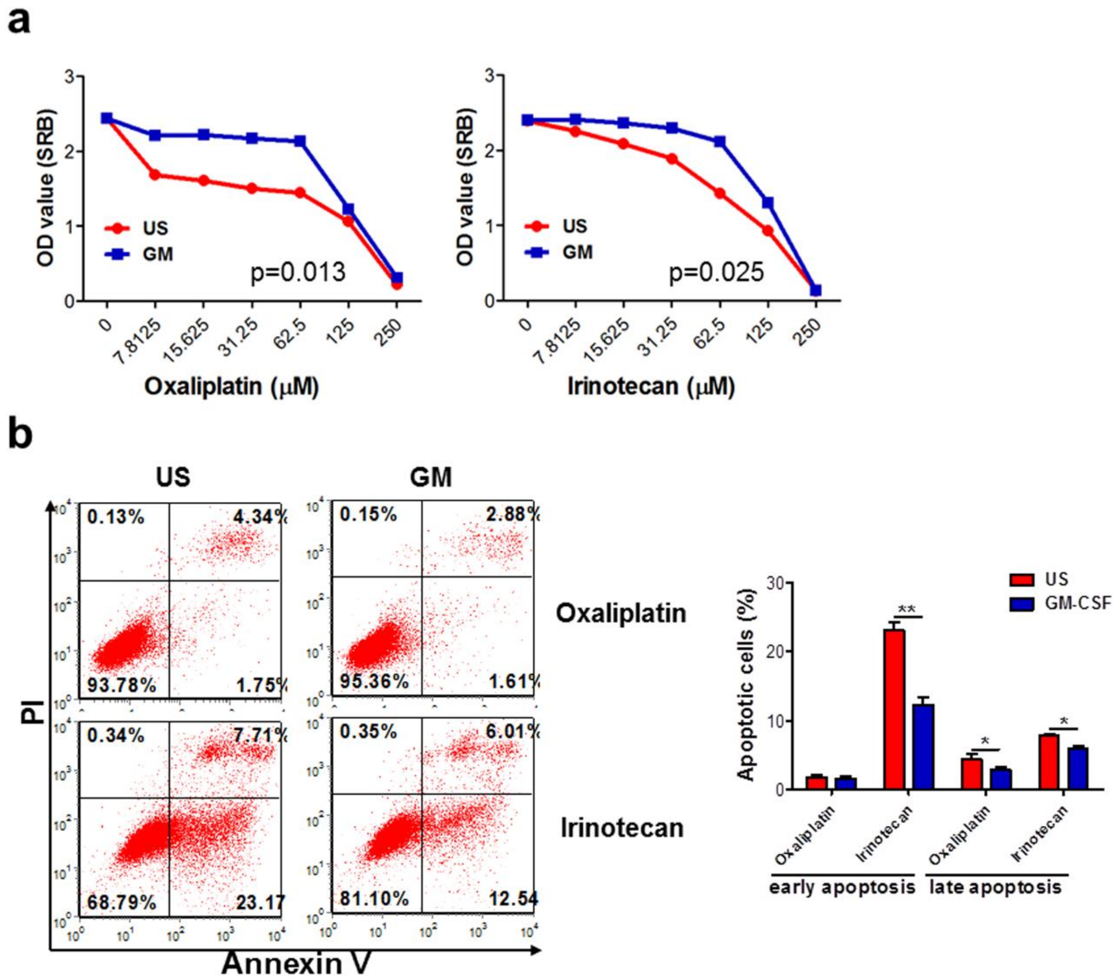
a
OD value (SRB)
US
GM
p=0.013
Oxaliplatin (μM)
p=0.025
Irinotecan (μM)
b
US
GM
PI
Annexin V
0.13%
4.34%
93.78%
1.75%
0.15%
2.88%
95.36%
1.61%
0.34%
7.71%
68.79%
23.17
0.35%
6.01%
81.10%
12.54
Oxaliplatin
Irinotecan
Apoptotic cells (%)
GM-CSF
early apoptosis
late apoptosis
